# Longitudinal Study of a Human Drug-Induced Model of Autoantibody to Cytoplasmic Rods/Rings following HCV Therapy with Ribavirin and Interferon-α

**DOI:** 10.1371/journal.pone.0045392

**Published:** 2012-09-24

**Authors:** Gerson Dierley Keppeke, Eunice Nunes, Maria Lucia Gomes Ferraz, Eduardo Antônio Benedito Silva, Celso Granato, Edward K. L. Chan, Luís Eduardo C. Andrade

**Affiliations:** 1 Rheumatology Division, Universidade Federal de São Paulo, São Paulo, Brazil; 2 Gastroenterology Division, Universidade Federal de São Paulo, São Paulo, Brazil; 3 Infectious Diseases Division, Universidade Federal de São Paulo, São Paulo, Brazil; 4 Immunology Division, Fleury Medicine and Health Laboratories, São Paulo, Brazil; 5 Department of Oral Biology, University of Florida, Gainesville, Florida, United States of America; University of North Carolina School of Medicine, United States of America

## Abstract

**Background:**

A novel pattern in the indirect immunofluorescence antinuclear antibody assay on HEp-2 cells (IIF-HEp-2) characterized by cytoplasmic rods and rings (RR) was reported in HCV patients, but stringent disease specificity studies and longitudinal analysis are lacking. We investigated the clinical significance of anti-RR in an HCV cohort with up to a 12-month treatment follow up.

**Methodology/Results:**

597 patients (342 HCV, 55 HCV/HIV, 200 non-HCV) were screened and titered for anti-RR. Serial samples were available from 78 of 176 treated and 27 of 166 untreated patients. Anti-RR was detected in 14.1% of 342 HCV patients, 9.1% of 55 HCV/HIV, 3.4% of 29 Hepatitis B, and none of 171 non-HCV (p<0.0001; HCV *versus* non-HCV). Anti-RR was present in 38% of 108 patients receiving interferon-α/ribavirin, but none in 26 receiving either interferon-α or ribavirin, or 166 untreated patients (p<0.0001). Other IIF-HEp-2 patterns were more frequently associated with interferon-α treatment alone (52.2%) as compared to interferon-α/ribavirin (25%), ribavirin alone (33.3%), and no therapy (26.5%). Anti-RR frequency was not associated with sex, age, ethnicity, HCV genotype or viral load. Anti-RR occurred only after initiation of treatment, beginning as early as 1 month (6%), but by the sixth month >47% tested positive for anti-RR. The anti-RR titer generally increased with sustained treatment and remained high in 53% of patients. After treatment, anti-RR titer was negative in 41%. Non-responders to HCV therapy were 77% in anti-RR-positive versus 64% in anti-RR-negative patients. Response to treatment was not associated with anti-RR titer or the dynamics of anti-RR reactivity during and after treatment.

**Conclusions:**

The exquisite association of anti-RR reactivity with combined interferon-α/ribavirin therapy in HCV patients represents a unique model for drug-induced autoantibody generation in humans as demonstrated by the fact that a significant fraction of patients who have anti-RR during therapy becomes anti-RR-negative after completion of therapy.

## Introduction

Autoantibodies with high avidity and in high concentration are usually detected in sera of patients with systemic autoimmune diseases, and indicate tolerance breakdown. The strict association of some autoantibodies with certain diseases has granted them the reputation of specific biomarkers [1], [2], [3], [4]. The identification of a novel autoantibody associated with a given disease may contribute to the understanding of its pathophysiology and may enrich the array of diagnostic tests for that disease [2]. The standard method for autoantibody screening is the indirect immunofluorescence assay on HEp-2 cells (IIF-HEp-2), historically known as the antinuclear antibody ANA test. However, a positive IIF-HEp-2 test is also observed in some patients with infectious and malignant diseases, as well as in up to 13% of healthy people [4], [5], [6].

A positive IIF-HEp-2 test has been reported in 7 to 50% of patients with HCV [7], [8], [9], [10], [11]. The few studies reporting on the immunofluorescence pattern of IIF-HEp-2 test in HCV patients have emphasized the nuclear fine speckled pattern and cytoplasmic fibrillar pattern [8], [9], [10], [12], [13]. Most IIF-HEp-2 reactivity in HCV patients is not associated with autoantibodies traditionally related to specific autoimmune diseases. However, a small fraction of HCV patients do present well characterized autoantibodies conventionally associated with autoimmune hepatitis, such as anti-LKM and anti-smooth muscle antibodies [14], [15], [16]. Anti-LKM antibody is classically associated with type 2 autoimmune hepatitis, but it has been observed in up to 10% of HCV patients, mostly males, and it appears to indicate mild liver histological and biochemical alterations in these patients [15], [17]. Anecdotal reports suggest that interferon-α therapy may worsen inflammatory liver activity and increase serum enzyme in LKM-reactive HCV patients [17], [18]. Anti-smooth muscle antibodies are directed mostly to the polymerized form of actin and are traditionally associated with type 1 autoimmune hepatitis, but they can also be observed in a small fraction of HCV patients, usually at a lower titer than in autoimmune hepatitis [16]. HCV patients presenting anti-smooth muscle autoantibodies appear not to differ from those without these autoantibodies concerning clinical profile and response to treatment [15], [19].

Recently a novel IIF-HEp-2 cytoplasmic pattern has been reported in HCV patients [20], [21], [22], [23], [24], [25]. It is characterized by a variable number of 3–10 µm long rods and 2–5 µm diameter rings spread throughout the cytoplasm. Accordingly, the novel IIF-HEp-2 pattern has been designated the “rods and rings” (RR) pattern. Interestingly, not all commercial HEp-2 slides are appropriate for the observation of the RR pattern. In fact, in many HEp-2 slides, the RR-positive serum samples yield a non-specific cytoplasmic speckled pattern or no relevant staining at all. This observation suggests that the target RR structures are evident only under special conditions. Alternatively it may be that the epitopes recognized by anti-RR antibodies are available only under special conditions. The RR structures seem to bear no relationship with the cytoskeleton, GW bodies, centrosomes, primary cilia structures, or “actin rockets” [22]. On the other hand, the RR structures resemble cytoplasmic structures previously reported in 1987 by Willingham, Richert, and Rutherford [22], [26]. These authors observed such structures in indirect immunofluorescence with a monoclonal antibody obtained by immunizing a Balb/c mouse with SR-Balb 3T3 cells. The putative antigen was named “nematin” due to the vermiform appearance of the stained structures and it could be detected in rat NRK and SR-NRK cell lines, in mouse Swiss 3T3, Balb 3T3, and SR-Balb cells, in human KB cells, and in bovine MDBK cells [26]. Unfortunately the monoclonal antibody, as well as the cell line, is no longer available (Mark Willingham, M.D., Wake Forest School of Medicine, personal communication).

At the moment it is not known why the IIF-HEp-2 RR pattern occurs only in a fraction of HCV patients. The present work aims to investigate how specific the anti-RR reactivity is to HCV and to evaluate possible relationships between the occurrence of the anti-RR reactivity and demographic, clinical, virological and therapeutic response characteristics of HCV patients.

## Materials and Methods

We studied samples from 597 patients, including 342 HCV patients, 55 HCV-HIV co-infected patients, and 200 miscellaneous patients (see below). Serum samples (n = 514) from 342 HCV patients were sequentially selected from the serum bank from the Gastroenterology Division at the Federal University of São Paulo (UNIFESP). All patients had a diagnosis of HCV hepatitis confirmed by the presence of anti-HCV antibodies, circulating HCV RNA, and biochemical and histological evidence of hepatitis. In addition, samples from 55 patients with HCV and HIV co-infection were analyzed. The control non-HCV group comprised serum samples from 200 patients with various hepatic and non-hepatic conditions not related to HCV, including systemic autoimmune rheumatic diseases (51 systemic lupus erythematosus, 36 systemic sclerosis, 8 polymyositis), multiple sclerosis (n = 7), and different liver diseases (29 hepatitis B, 69 autoimmune hepatitis). Diagnostic classification of patients complied with the internationally accepted classification criteria [27], [28], [29], [30], [31], [32], [33], [34], [35]. All samples were obtained from 1998 to 2008 and were stored at −20°C. The informed consent form was signed by patients currently followed at the institution. For a small fraction of patients not available at the present time, serum bank historical samples were used for assays regularly ordered for their clinical conditions. The study has been approved by the institution's Ethics Committee.

Among the 342 HCV patients, 176 had been treated for HCV and had available samples during and after treatment; 166 HCV patients had not been treated. Serial samples (2 to 5) were available from 105 patients, namely 27 untreated patients and 78 treated patients with samples before, during, and after treatment. All co-infected patients had serial samples (2 to 4) before, during and after the treatment. Clinical data were obtained from an electronic data bank based on a structured protocol with detailed parameters regarding clinical, biochemical, therapeutic response, and virological aspects. The number of patients analyzed for different parameters varied because not all parameters were available for all patients. For the analysis of the occurrence of the anti-RR reactivity as a function of treatment, only patients for whom treatment had been indicated were included. HCV viral load was determined by quantitative PCR (Amplicor®, Roche, Basel, Switzerland) with sensitivity of 600 IU/mL and linearity up to 850,000 IU/mL. Virologic response was determined in 125 patients who had consistent monitoring of HCV viral load within the 6 months following completion of treatment. Patients with consistently negative PCR for HCV up to 6 months after the end of treatment were classified as having developed a sustained virologic response. Patients who never had undetectable serum HCV levels and those who had undetectable HCV at the end of treatment, but presented detectable HCV within 6 months after treatment suspension, were classified as non-responders.

All samples were processed for IIF with HEp-2 slides from Euroimmun (Lübeck, Germany) and INOVA (San Diego, CA, USA) at a screening dilution of 1/80 according to standard indirect immunofluorescence protocol. Representative samples were also tested in other HEp-2 slide brands: Hemagen (Virgo®, Columbia, Maryland, USA), MBL Bion (Des Plaines, Illinois, USA), and Immuno Concepts (Sacramento, CA, USA). In addition, homemade slides were prepared by cultivating HEp-2 cells (ATCC #CCL-23; Virginia, USA) on round coverslips with Dulbecco's Modified Eagle Medium (DMEM) in 10% Fetal Calf Serum (FCS) and 1% Gibco Antibiotic/Antimycotic reagent (Penicillin G, Streptomycin, amphotericin) at 37°C and 5% CO_2_. In some experiments, cells were treated with 2 mM ribavirin (Farmanguinhos Laboratory, Rio de Janeiro, Brazil) and incubated for 24 h. Cells were fixed with methanol for 8 min and acetone for 2 min at −20°C.

Briefly, samples were diluted at 1/80 in 0.15 M NaCl, 10 mM phosphate buffered saline, pH 7.4 (PBS), and incubated with HEp-2 cells for 30 min at room temperature in a moist chamber. After washing twice in PBS for 10 min, cells were incubated with fluorescein isothiocianate-labeled anti-human immunoglobulin (IgG heavy and light chains) goat immunoglobulin for another 30 min period in the dark. After washing twice as before, slides were assembled with buffered glycerol pH 9.5 and cover slips. Autoantibody titer was determined by testing successive 2-fold dilutions of the serum up to 1/10,240. Analysis was performed by two independent expert observers (GDK & LEA) using an Olympus BX 50 microscope under 400× magnification. Images were captured using a Carl Zeiss Axio-Imager M2 microscope.

Categorical variables, such as sex, age group, and IIF patterns, were analyzed using the Chi-square test and the Fisher exact test when appropriate. Quantitative and semi-quantitative parameters with normal distribution were analyzed by ANOVA, and those with non-Gaussian distribution were analyzed using the Kruskal-Wallis test. All data were analyzed using SPSS for Windows 19.0. p values less than 0.05 were considered significant.

## Results

**Figure 1:**
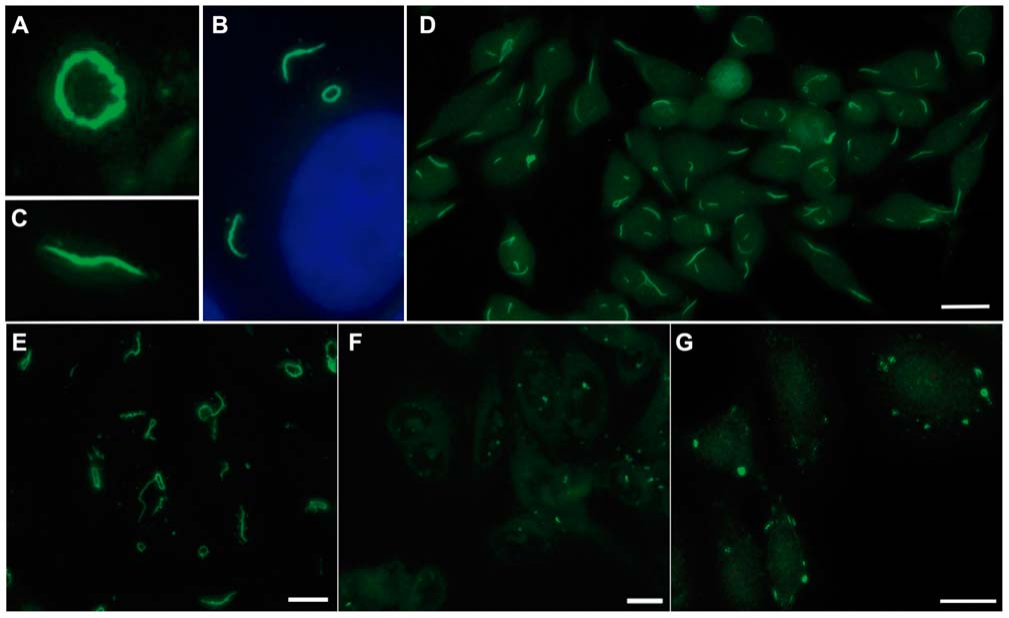
IIF-HEp-2 rods and rings pattern in different slide brands. Indirect immunofluorescence with a representative anti-RR-positive HCV patient sample diluted 1/80. A, B, and C, slides from Euroimmun at ×1,000 magnification. (A) Ring conformation example; (B) Rod conformation example; (C) Cell with both conformations, depicting the cytoplasmic localization underscored by DAPI staining of the nucleus. (D) Classical RR pattern in INOVA slides, depicting a variable number of 3–10 µm long rods and 2–5 µm diameter rings in the cytoplasm. (E) Typical RR configuration in home-made slides with HEp-2 cells treated with 2 mM ribavirin for 24 h. Non-specific cytoplasmic dots in non-treated home-made HEp-2 slides (F) and in Bion HEp-2 slides (G), respectively. White bar: 20 µm.

RR reactivity was readily recognized on Euroimmun and INOVA HEp-2 slides but either non-specific cytoplasmic dots or no relevant staining was observed with other commercial or homemade HEp-2 slides (Figure 1). In contrast, typical RR structures were readily apparent in HEp-2 cells treated with ribavirin (Figure 1E). Anti-RR reactivity was detected in sera of 57 of the 597 patients studied (9.5%), including 56 of the 397 with Hepatitis C (14.1%), 1 of 29 with Hepatitis B (3.4%), and none of 171 patients without any form of viral hepatitis. Thus, anti-RR reactivity was strongly associated with HCV (p<0.0001; Fisher's exact test between HCV and non-HCV controls). There was no significant difference in anti-RR reactivity between 51 of the 342 patients (14.9%) with HCV infection alone and 5 of the 55 patients (9.1%) with HCV/HIV co-infection (p = 0.301; Fisher's exact test). Except for the single patient with Hepatitis B, no sample from patients with other hepatic diseases and systemic autoimmune diseases presented anti-RR reactivity. In contrast, other IIF-HEp-2 patterns were observed in variable proportions in all groups of patients. The nuclear fine speckled pattern was the dominant pattern in most patients, except for HCV patients in whom the RR and nuclear fine speckled patterns displayed a similar frequency (Table 1).

**Table 1:** Distribution of patients with HCV and other hepatic and non-hepatic diseases according to the presence of anti-RR and other IIF-HEp-2 patterns.

	IIF-HEp-2 patterns*									
Disease groups	NFS	NHo	NCS	NQH	Cen	Cyt	No	RR	Misc	Overall IIF-HEp-2 reactivity
**Hepatitis C (n = 342)**	55	5	-	9	-	7	14	**51** **	26	145 (42%)
**Hepatitis B (n = 29)**	4	-	-	-	-	-	-	**1**	-	5 (17%)
**Autoimmune Hepatitis (n = 69)**	21	3	1	16	1	1	2	**-**	14	49 (70%)
**Systemic Lupus Erythematosus (n = 51)**	25	16	4	1	-	1	5	**-**	2	46 (90%)
**Systemic Sclerosis (n = 36)**	9	4	5	3	8	-	6	**-**	6	32 (9%)
**Polymyositis (n = 8)**	5	-	-	2	1	-	1	**-**	-	8 (100%)
**Multiple Sclerosis (n = 7)**	1	-	-	-	1	-	-	**-**	-	2 (29%)
**HCV and HIV co-infection (n = 55)**	7	-	1	-	-	4	5	**5**	5	26 (47%)

* IIF-HEp-2 patterns were based on observation using INOVA and Euroimmun slides.

** Seven patients presented anti-RR reactivity associated with other pattern. We include these patients only in the anti-RR positive group.

NFS: Nuclear Fine Speckled; NHo: Nuclear Homogeneous; NCS: Nuclear Coarse Speckled; NQH: Nuclear Quasi Homogeneous; Cen: Centromere; Cyt: Cytoskeleton; No: Nucleolar; RR: Rods and Rings; Misc: Miscellaneous patterns: Scl-70-like pattern, RNA polymerase I pattern, multiple nuclear dots, nuclear envelope, nuclear matrix, dense fine speckled pattern.

**Figure 2:**
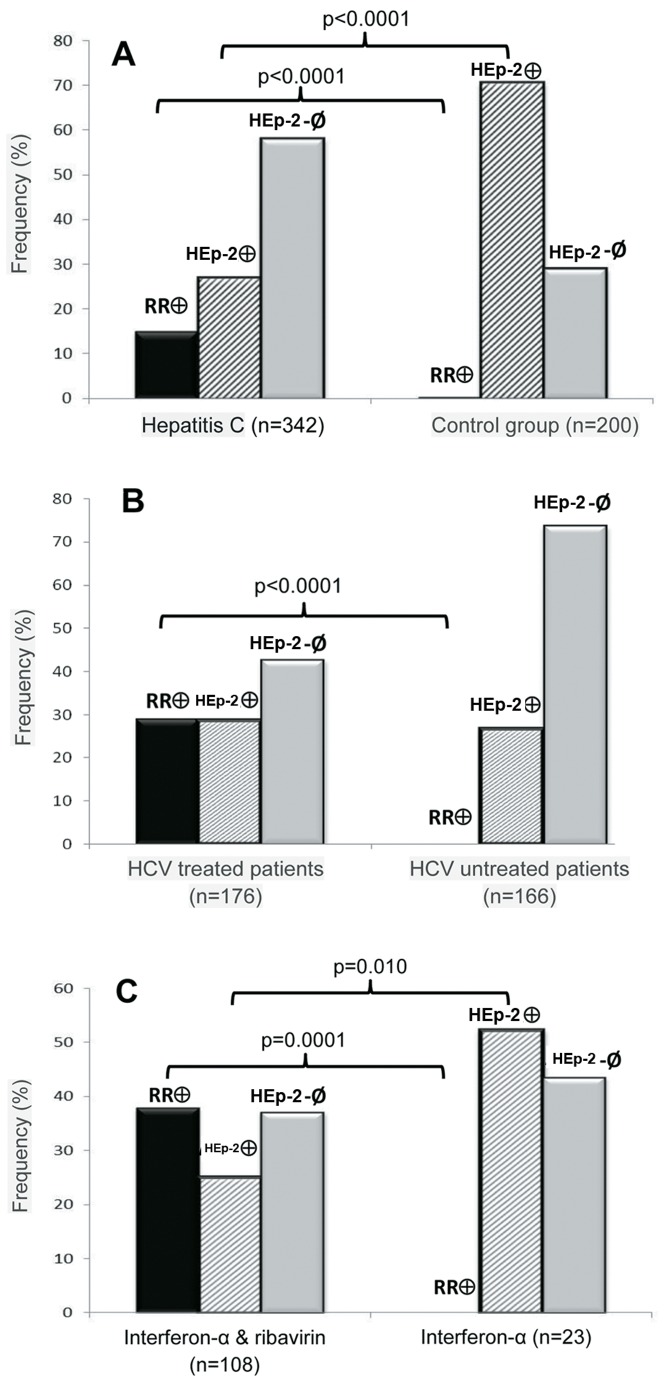
Antibodies to rods and rings are predominantly observed in HCV patients undergoing treatment with interferon-α plus ribavirin. Comparison of IIF-HEp-2 patterns in (A) Patients with HCV and the control group (systemic lupus erythematosus, systemic sclerosis, polymyositis, multiple sclerosis, hepatitis B, autoimmune hepatitis); (B) Treated and untreated HCV patients; and (C) HCV patients treated with interferon-α plus ribavirin and those receiving only interferon-α. Black bars: Anti-RR-reactive; gray-lined bars: other IIF-HEp-2 patterns; gray solid bars: IIF-HEp-2-negative.

Among HCV patients, anti-RR reactivity was clearly associated with treatment with ribavirin and interferon-α. Treatment with interferon-α and/or ribavirin had been administered to 176 HCV patients, while 166 HCV patients received no treatment. Anti-RR reactivity was detected in 51 treated patients (29%), but in none of the untreated patients (p<0.0001; Fisher's exact test). In contrast, other IIF-HEp-2 patterns were observed in 50 treated patients (28.4%) and in 44 untreated patients (26.5%) (p = 0.598). Information regarding the precise therapeutic regimen was available for 134 patients: 108 were receiving interferon-α plus ribavirin, 23 were receiving only interferon-α, and 3 patients were receiving only ribavirin. Anti-RR reactivity was detected in 41 patients treated with interferon-α plus ribavirin (38%), but in none of the patients treated with either one of the two drugs (p = 0.0001; Fisher's exact test, Figure 2). In contrast, other IIF-HEp-2 patterns were more frequent in patients receiving only interferon-α (52.2%) than in those treated with interferon-α plus ribavirin (25%) and those treated only with ribavirin (33.3%) (p = 0.01) (Figure 2). Accurate information on treatment was available for 21 patients with HIV/HCV co-infection. Anti-RR reactivity was detected in 5 of the 20 patients treated with interferon-α plus ribavirin (20%), but not in the single patient treated only with interferon-α.

**Figure 3:**
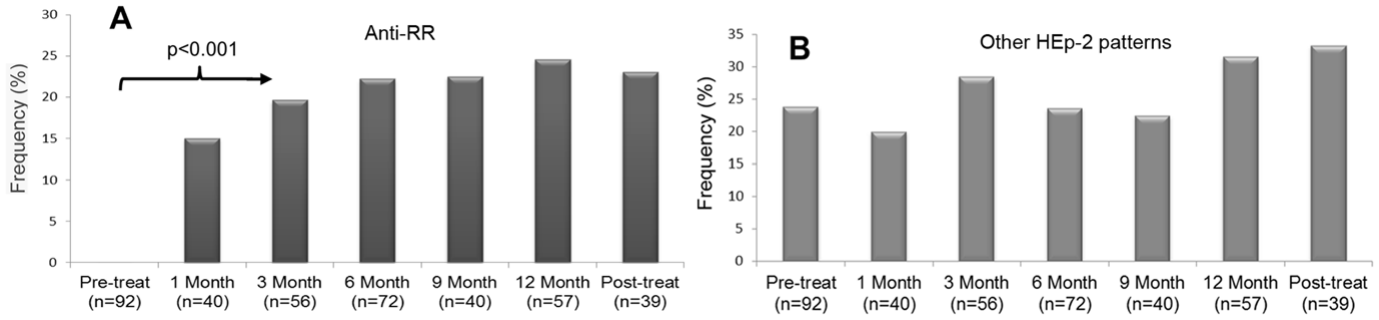
Anti-RR reactivity occurs at varying intervals after the beginning of treatment. Samples from HCV patients were obtained at baseline, at successive months after beginning of treatment with interferon-α plus ribavirin, and 6–12 months after treatment discontinuation. (A) Anti-RR was absent in samples obtained at the beginning of treatment and there was a clear trend for increasing frequency in samples obtained along several time points during and after treatment; the frequency rate differed between the pre-treatment bleed and other time points, but not among the several bleeds after the beginning of treatment (p = 0.705). (B) The frequency of other IIF-HEp-2 patterns was equivalent in samples obtained before the beginning of treatment and in those obtained along and after the treatment (p = 0.727).

The apparent association of anti-RR reactivity and therapy with interferon-α plus ribavirin was further strengthened by the analysis of serial samples of RR-positive patients before and after the beginning of treatment. For this analysis, anti-RR reactivity was compared in samples obtained before and each month after the beginning of treatment, and also in samples obtained after treatment had been discontinued. As observed in Figure 3A, all pre-treatment samples were anti-RR negative, and an increasing frequency of anti-RR was observed during the several months of treatment. Even after discontinuation of therapy with interferon-α plus ribavirin, some anti-RR reactivity was maintained when evaluated up to one year later (Figure 3A). All time-point groups after initiation of treatment showed higher frequency of anti-RR reactivity compared to pre-treatment samples (p<0.001; Pearson Chi-square test). In contrast, there was no difference in the frequency of other IIF-HEp-2 patterns in samples obtained before and after initiation of therapy with interferon-α plus ribavirin (Figure 3B, p = 0.727). In addition, no anti-RR reactivity was observed in the serial samples from 27 untreated patients. Treated patients showed no statistical difference regarding the frequency of anti-RR during the months on treatment (Figure 3A, p = 0.705).

**Figure 4:**
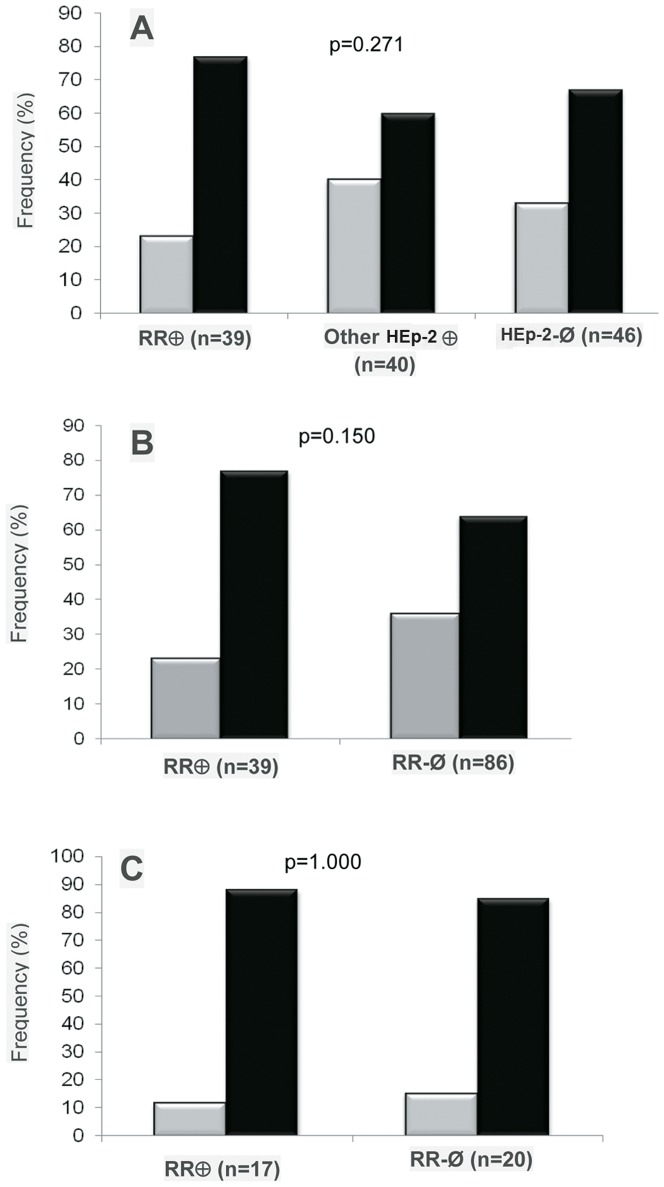
Anti-RR is not related to sustained virologic response. The frequency of samples with RR IIF-HEp-2 pattern, other patterns and those with a negative test was equivalent in patients exhibiting sustained virologic response and those classified as non-responders. (A and B) HCV patients in general; (C) HCV patients with genotype 1 and viral load above 100,000 IU/mL. Gray bars: sustained virologic response; black bars: non-responder patients.

There was consistent information on the profile of therapeutic response for 125 patients, for whom no association was observed between the appearance of anti-RR and therapeutic response. The proportion of non-responders was equivalent (p = 0.271; Pearson Chi-square test) in the 39 patients with anti-RR reactivity (77%), 40 patients with other IIF-HEp-2 patterns (60%), and 46 patients with negative IIF-HEp-2 tests (67%, Figure 4A and 4B). In order to examine possible influences of HCV genotype and viral load on the pattern of response to treatment, the analysis was repeated solely including patients infected with genotypes 1A, 1B, and 1C (most prevalent HCV genotype in the region [36], [37], [38]), and with baseline viral loads equal to or greater than 100,000 IU/mL at the beginning of treatment. This analysis comprised 17 patients with anti-RR and 20 patients presenting other IIF-HEp-2 patterns or negative for the IIF-HEp-2 test. Fifteen of the anti-RR positive patients (88%) and 17 of the anti-RR negative patients (85%) were classified as non-responders (p = 1.000) (Figure 4C). Next the titer of anti-RR antibodies was analyzed according to the profile of therapeutic response. For this analysis, the highest titer in serial samples for each patient was considered. The titer of anti-RR reactivity for all anti-RR-positive patients (n = 57) varied from 1/80 to ≥1/10240 with a median of 1/1280: 12 (21%) of the samples had low titer (1/80 and 1/160), 5 (9%) had medium titer (1/320), and 40 (70%) had high titer (≥1/640). Among the 39 patients for whom information about therapeutic response was available, anti-RR titer varied from 1/160 to ≥1/2,560. Patients presenting sustained virologic response had titers of 1/160 to 1/2,560, with a median of 1/1,280. Non-responders had titers varying from 1/80 to 1/2,560, with a median of 1/1,280. There were 26 patients with high titer (≥1/640) and 11 patients with low titer anti-RR reactivity (≤1/160). Two patients had intermediate titers of 1/320. Among patients with high titer anti-RR reactivity, 19 (73%) were non-responders and 7 (27%) presented sustained virologic response. Among those with low titer anti-RR reactivity, 9 (82%) were non-responders and 2 (18%) presented sustained virologic response (p = 0.694; Fisher's exact test).

**Figure 5:**
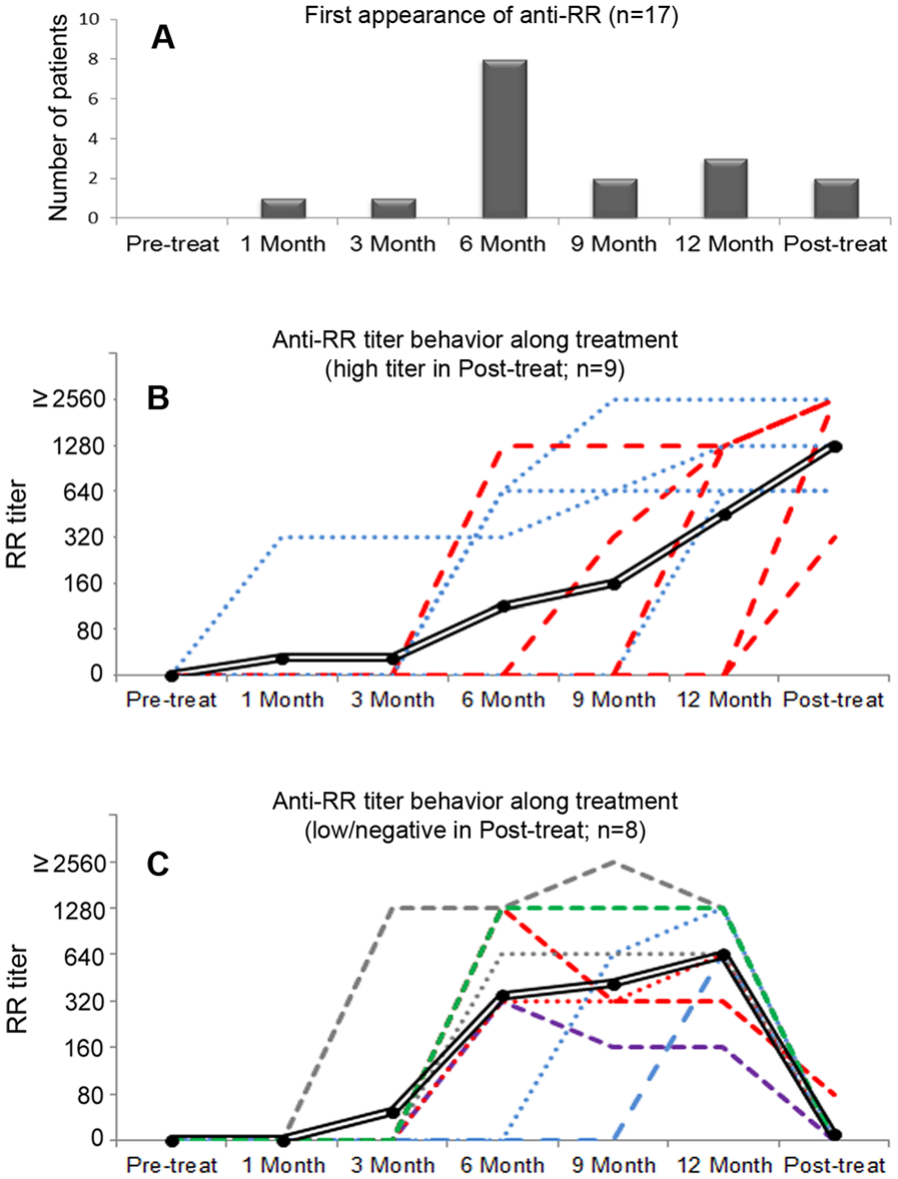
Anti-RR first appearance and titer behavior during and after treatment discontinuation. (A) Time for first appearance of anti-RR reactivity. For those 17 patients included in this analysis, 8 (47%) presented anti-RR reactivity at the sixth month of treatment and two produced their first anti-RR sample after treatment discontinuation (in red dashed lines in panel B). (B) Nine patients kept or increased the titer of anti-RR after treatment discontinuation. Titer showed a trend for increasing along anti-HCV treatment and after treatment discontinuation. Double black line = overall trend; red dashed line = anti-RR titer increased after treatment discontinuation (n = 5); blue dots line = anti-RR titer was stable after treatment discontinuation (n = 4). (C) In eight patients, anti-RR reactivity became negative (color dashed and dots lines, n = 7) or dropped down to 1/80 (red dashed line, n = 1) after treatment discontinuation. Double black line = overall trend. 0 = negative. Dilution was from 1/80 into 1/2560 for this analysis.

For 17 anti-RR-positive patients receiving the first round of treatment, there were at least four sequential samples, including the pre-treatment sample. All these samples were blind and simultaneously processed for the determination of anti-RR titer. For all patients, the baseline pre-treatment sample was negative and anti-RR reactivity started at low titer and increased as the treatment progressed (Figure 5). The temporal appearance of anti-RR at the beginning, during, and after therapy was further analyzed. There was an overall trend for increase in anti-RR titer during the therapy (Figure 5B and C), but there was considerable heterogeneity in the tempo and steadiness of anti-RR titer among different patients. There was also heterogeneity regarding the interval from the beginning of therapy and the first positive result for anti-RR reactivity. Only two patients (12%) showed anti-RR reactivity before 6 months of treatment. In 10 of the 17 patients (59%), anti-RR reactivity first appeared before or at the sixth month of treatment (early appearance), and 7 patients (41%) showed anti-RR reactivity at or after the 9^th^ month of therapy (late appearance; Figure 5A and B). Sustained virologic response was observed in 3 of the patients with early anti-RR appearance (30%) and in 3 of those with late anti-RR appearance (43%) (p = 0.643; Fisher's exact test). In the six months after treatment discontinuation, patients showed heterogeneous anti-RR behavior: nine patients kept or increased their previous titer, and eight patients became negative or dropped in titer to 1/80 (Figure 5B and C). No relationship was apparent between anti-RR titer after discontinuation of treatment and the profile of therapeutic response, since an unsatisfactory therapeutic response was observed in 7 (77%) of those patients who maintained high titer anti-RR reactivity and in 5 (62.5%) of those who experienced an accentuated drop in anti-RR reactivity.

Next we investigated the possible influence of demographic variables, HCV genotype, and HCV viral load on the occurrence of anti-RR reactivity in patients treated with interferon-α plus ribavirin. As depicted in Table 2, patients divided according to IIF-HEp-2 patterns (negative, RR, and other IIF-HEp-2 patterns) did not differ according to age (p = 0.199; ANOVA), sex (p = 0.762; Pearson Chi-square test), or ethnic group (p = 0.417; Pearson Chi-square test). There was also no difference in the average duration of Hepatitis C diagnosis (p = 0.515; ANOVA). The predominant HCV genotypes were 1A (47%) and 1B (38%), and there was no difference in genotype distribution according to the IIF-HEp-2 patterns (p = 0.679; Pearson Chi-square test). As depicted in Table 2, HCV viral load was similar in anti-RR-reactive patients (361,222±64,842), IIF-HEp-2-negative patients (348,492±58,816), and patients with other IIF-HEp-2 patterns (390,194±66,071; p = 0.776; Kruskal-Wallis test).

**Table 2:** Demographic data, time of HCV diagnosis, HCV genotype, and HCV viral load in HCV patients according to the presence of anti-RR and other IIF-HEp-2 patterns* .

		Anti-RR positive patients	IIF-HEp-2 negative patients	Other IIF-HEp-2 patterns	Statistical analysis
Age in years (mean ± DP) (n = 107)		51.5±1.9	48.6±1.4	53.1±2.2	p = 0.199
Sex (%) (n = 136)	Male (n = 82)	31 (37.8%)	29 (35.4%)	22 (26.8%)	p = 0.762
	Female (n = 54)	20 (37%)	17 (31.5%)	17 (31.5%)	
Ethnicity (%) (n = 89)	White (n = 54)	22 (40.7%)	17 (31.5%)	15 (27.8%)	p = 0.417
	Mulatto (n = 21)	6 (28.6%)	11 (52.4%)	4 (19%)	
	Black (n = 14)	3 (21.4%)	6 (42.9%)	5 (35.7%)	
Length of diagnosis in months (mean ± DP) (n = 70)		19.5±2.5	18.2±1.5	16.1±2.1	p = 0.515
HCV Genotype (%) (n = 81)	1A (n = 38)	13 (34.2%)	17 (44.7%)	8 (21.1%)	p = 0.679
	1B (n = 31)	14 (45.2%)	12 (38.7%)	5 (16.1%)	
	Others (1C, 3A e 3B) (n = 12)	3 (25%)	8 (66.7%)	1 (8.3%)	
HCV viral load (mean ± DP) (n = 72)		361,222±64,842	348,492±58,816	390,194±66,071	p = 0.776

* The total number of subjects analyzed varied for each parameter according to the availability of consistent information.

## Discussion

Anti-RR reactivity has been reported in the last few years as a peculiar IIF-HEp-2 pattern observed with samples from HCV patients [20]. Preliminary work has indicated that this novel IIF-HEp-2 pattern occurs predominantly in HCV patients undergoing therapy with interferon-α [21], [22] It has also been demonstrated that the RR pattern is observed solely when using certain HEp-2 slide brands. This observation implies that the RR structures are not readily available under regular circumstances and that they must be induced by particular cultivation and fixation conditions. In fact that was observed with the in vitro treatment of HEp-2 cells with ribavirin. Another possibility could be that the recognized epitopes may not be naturally found under physiologic conditions. However, the observation that anti-RR samples elicit an array of cytoplasmic patterns – ranging from non-specific dots to fine speckles – in other HEp-2 slide brands and in homemade HEp-2 slides suggests that the recognized epitopes are indeed naturally available in less characteristic arrangements as compared to the RR structures.

The present study focused solely on the clinical aspects of this particular immune response in an attempt to understand what factors are involved in its occurrence. We were able to confirm that antibodies associated with the RR pattern are strongly associated with Hepatitis C. In addition, we could demonstrate that the occurrence of anti-RR reactivity is promoted by the combined therapy with interferon-α plus ribavirin and that its frequency increases with the length of treatment, but it was not observed in patients treated with either one of these two drugs alone. No relationship could be recognized between anti-RR reactivity and demographic parameters, duration of HCV diagnosis, pattern of response to treatment, HCV genotype, or HCV viral load. In addition, the existence of co-infection with HIV did not affect the occurrence of anti-RR reactivity in HCV patients and its relationship with combined interferon-α plus ribavirin therapy.

The association of anti-RR reactivity and combined therapy with interferon-α plus ribavirin is an interesting finding. It is well known that interferon-α is able to stimulate innate immunity and has a general adjuvant effect on the immune system. Interferon-α is a type 1 interferon produced by B and T lymphocytes, NK cells, monocytes, and virus-infected cells. After binding to surface receptors, it activates Janus-activated kinase-1 (Jak1) and tyrosine kinase 2 (Tyk2) that phosphorylate the signal transducers and activators of transcription proteins (STAT1 and STAT2) [39], [40]. Activated STAT1 and STAT2 reach the nucleus where they bind to the interferon regulator factor 9 (IRF-9) to form a complex that will promote the transcription of a series of interferon-stimulated genes (ISG). The products of ISG block the synthesis of viral proteins by inhibiting the Eukaryotic Initiation Factor eIF2 and therefore contribute to the establishment of an antiviral intracellular environment [41], [42], [43], [44]. In addition, type 1 interferons act indirectly against the virus by means of stimulating the immune response. Generation of antibodies and autoantibodies seems to be boosted by persistently increased concentration of type 1 interferon. In fact, systemic lupus erythematosus, possibly the disease with the highest occurrence of autoantibodies, has been demonstrated to exhibit high levels of circulating type 1 interferon and increased expression of ISG [45], [46]. In the present study we could confirm the autoantibody-inducing ability of interferon-α by the observation that 52% of HCV patients receiving just interferon-α, but only 26.2% of untreated patients, presented a positive IIF-HEp-2 test. Interestingly, none of these HCV patients exclusively receiving interferon-α developed antibodies associated with the IIF-HEp-2 RR pattern. This is in accordance with the observation that anti-RR reactivity was also not observed in patients with systemic lupus erythematosus.

The fact that isolated interferon-α therapy was not associated with anti-RR reactivity appeared to suggest that ribavirin would be required for this peculiar type of antibody response. Ribavirin is a synthetic guanosine analog with direct action against RNA and DNA viruses, possibly through inhibition of virus-dependent polymerases. Similarly to guanosine, ribavirin is phosphorylated within the cell into monophosphate (RMP), diphosphate (RDP), and triphosphate (RTP) ribavirin. The interaction of RTP with cellular and viral enzymatic machineries has a host of effects, including the inhibition of inosine monophosphate dehydrogenase with depletion of guanosine triphosphate (GTP) necessary for viral RNA synthesis [47], direct inhibition of HCV RNA polymerase NS5B RdRp [48], [49], and induction of a high rate of viral RNA mutagenesis resulting in a decline in the number of viable viral copies [50], [51]. However, these effects vary depending on the particular virus and RTP has been shown to be a weak inhibitor for viral RNA polymerases related to HCV [52]. Ribavirin also enhances hepatocyte gene response to peginterferon [53]. Accordingly, ribavirin on its own is effective for only a minority of HCV patients, but it is a valuable adjuvant in the therapy with interferon-α [54].

Curiously, the few patients receiving only ribavirin did not present anti-RR reactivity. Although one cannot rule out that ribavirin alone can trigger anti-RR, it appears that the occurrence of anti-RR reactivity is strongly favored by the combined effects of interferon-α and ribavirin. It is possible that interferon-α is required to stimulate the occurrence of anti-RR reactivity apparently driven by ribavirin. Recent reports suggested that possible targets of anti-RR reactivity include CTP synthase and Inosine-5′-monophosphate dehydrogenase-2 (IMPDH2), an enzyme involved in the generation of GTP and inhibited by ribavirin [23], [24], [25]. The observation that HEp-2 cells treated in vitro with ribavirin were an adequate substrate to yield typical RR structures reinforces the idea that ribavirin is involved in the particular spatial rearrangement of these putative autoantigens and this may contribute to the generation of autoantibodies against these enzymes. In this sense it is particularly exciting that three recent independent studies have demonstrated that CTP synthase may polymerize under special conditions and present as filamentous cytoplasmic structures reminiscent of the rods of RR structures [55], [56], [57], [58].

Despite the evidence implying interferon-α and ribavirin in the induction of anti-RR reactivity, it is important to emphasize that one Hepatitis B patient treated with lamuvidine also presented the characteristic anti-RR reactivity with titer >1/1280. Unfortunately the baseline serum sample for this patient was not available. However, the titer of anti-RR antibodies after the patient's first month of treatment was at 1/80 and subsequently increased along with treatment up to 1/1,280 at the twelfth month. This observation may suggest that lamuvidine may also contribute to exposure of RR-related immunogenic epitopes and that liver inflammation per se may also exert adjuvant effects toward the occurrence of this particular antibody response.

## Conclusions

In conclusion, the present study independently confirmed the strict association of the IIF-HEp-2 RR pattern with HCV and extended this association to HCV/HIV co-infection. In addition we were able to narrow down the trigger for anti-RR reactivity to the combined interferon-α plus ribavirin therapy. In contrast, no association was detected with demographic parameters, duration of diagnosis, response to treatment, HCV genotype, or HCV viral load. Despite the clear association of anti-RR reactivity and combined interferon-α plus ribavirin therapy, it is striking that the majority (62%) of patients under this therapy showed no anti-RR reactivity. This observation indicates that other factors must play a role in determining the occurrence of this peculiar antibody response. Future studies should address potential candidate factors, such as the MHC genotype, the severity and extent of liver inflammatory reaction, and genetic polymorphism related to enzymes involved in the processing of ribavirin or to enzymes targeted by ribavirin.
